# Bis(ethyl­enediamine-κ^2^ *N*,*N*′)(nitrato-κ^2^ *O*,*O*′)cobalt(III) hydroxide nitrate

**DOI:** 10.1107/S160053680903219X

**Published:** 2009-08-19

**Authors:** Ji-Bo Zhang, Xiao-Shu Qu

**Affiliations:** a Jilin Provincial Universities Engineering Research Center for Chemical Separation Technology, Jilin Institute of Chemical Technology, Jilin 132022, People’s Republic of China; b Department of Chemistry and Pharmaceutical Engineering, Jilin Institute of Chemical Technology, Jilin 132022, People’s Republic of China

## Abstract

The Co ion in the title salt, [Co(NO_3_)(H_2_NCH_2_CH_2_NH_2_)_2_](OH)(NO_3_), has oxidation state + III and is coordinated by four N atoms from two ethyl­enediamine mol­ecules and two O atoms from a nitrate anion in a distorted octa­hedral geometry. The charge of the complex cation is balanced by a hydroxide anion and a nitrate anion. The cations and anions are connected by N—H⋯O and O—H⋯O hydrogen bonds, resulting in a three-dimensional supra­molecular framework. There are two independent ion pairs with similar configurations in the unit cell. Both uncoordinated nitrate counter-anions are disordered.

## Related literature

For diethyl­enediamine-chelated Co(III) complexes with Cl^−^ or SO_4_^2−^ as the second ligand, see: Anderson *et al.* (1977 ▶); Niederhoffer *et al.* (1986 ▶); Sharma *et al.* (2006*a* ▶,*b* ▶,*c* ▶). For comparison Co—N and Co—O distances, see: Bruggemann & Thewalt (1994 ▶); Sharma *et al.* (2005 ▶). 
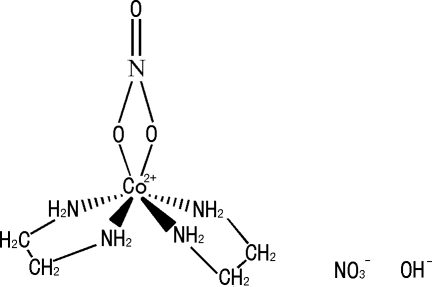


## Experimental

### 

#### Crystal data

[Co(NO_3_)(C_2_H_8_N_2_)_2_](OH)(NO_3_)
*M* *_r_* = 320.17
Monoclinic, 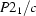

*a* = 9.5212 (13) Å
*b* = 23.163 (3) Å
*c* = 12.6473 (13) Å
β = 118.491 (7)°
*V* = 2451.4 (5) Å^3^
*Z* = 8
Mo *K*α radiation
μ = 1.44 mm^−1^
*T* = 296 K
0.38 × 0.34 × 0.28 mm

#### Data collection

Bruker APEX CCD area-detector diffractometer
Absorption correction: multi-scan (*SADABS*; Sheldrick, 1996 ▶) *T*_min_ = 0.585, *T*_max_ = 0.660
13327 measured reflections
4801 independent reflections
3864 reflections with *I* > 2σ(*I*)
*R*_int_ = 0.080

#### Refinement

*R*[*F* ^2^ > 2σ(*F* ^2^)] = 0.043
*wR*(*F* ^2^) = 0.116
*S* = 1.00
4801 reflections
379 parameters
156 restraints
H-atom parameters constrained
Δρ_max_ = 0.72 e Å^−3^
Δρ_min_ = −0.94 e Å^−3^

### 

Data collection: *SMART* (Bruker, 1997 ▶); cell refinement: *SAINT* (Bruker, 1999 ▶); data reduction: *SAINT*; program(s) used to solve structure: *SHELXS97* (Sheldrick, 2008 ▶); program(s) used to refine structure: *SHELXL97* (Sheldrick, 2008 ▶); molecular graphics: *SHELXTL-Plus* (Sheldrick, 2008 ▶); software used to prepare material for publication: *SHELXL97*.

## Supplementary Material

Crystal structure: contains datablocks global, I. DOI: 10.1107/S160053680903219X/ng2611sup1.cif

Structure factors: contains datablocks I. DOI: 10.1107/S160053680903219X/ng2611Isup2.hkl

Additional supplementary materials:  crystallographic information; 3D view; checkCIF report

## Figures and Tables

**Table 1:** Hydrogen-bond geometry (Å, °)

*D*—H⋯*A*	*D*—H	H⋯*A*	*D*⋯*A*	*D*—H⋯*A*
O13—H13⋯O10	0.98	1.97	2.906 (8)	158
O14—H14⋯O8	0.87	2.04	2.78 (3)	143
N1—H1*A*⋯O4^i^	0.90	2.17	3.032 (3)	160
N1—H1*B*⋯O5^ii^	0.90	2.19	2.995 (3)	148
N2—H2*A*⋯O14^iii^	0.90	2.00	2.888 (4)	168
N2—H2*B*⋯O8^iv^	0.90	2.25	3.045 (19)	148
N3—H3*A*⋯O6^ii^	0.90	2.10	2.986 (3)	169
N3—H3*B*⋯O9^i^	0.90	2.33	3.14 (2)	150
N4—H4*A*⋯O3^v^	0.90	2.22	3.004 (3)	145
N4—H4*B*⋯O12	0.90	2.26	3.071 (14)	150
N6—H5*A*⋯O12^i^	0.90	2.10	2.973 (14)	163
N6—H5*B*⋯O13^vi^	0.90	2.53	3.258 (4)	138
N7—H6*A*⋯O2^v^	0.90	1.99	2.881 (3)	168
N7—H6*B*⋯O1	0.90	1.98	2.873 (3)	169
N8—H7*A*⋯O7	0.90	2.18	2.988 (7)	149
N8—H7*B*⋯O6^vii^	0.90	2.01	2.887 (3)	163
N9—H8*A*⋯O11^viii^	0.90	2.29	3.088 (9)	147
N9—H8*A*⋯O12^i^	0.90	2.58	3.08 (2)	115
N9—H8*B*⋯O3	0.90	2.19	3.065 (3)	163

Symmetry codes: (i) 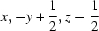; (ii) 
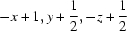
; (iii) 
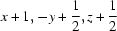
; (iv) 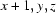; (v) 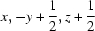; (vi) 
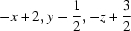
; (vii) 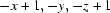; (viii) 
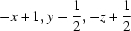
.

**Fig. 1.**
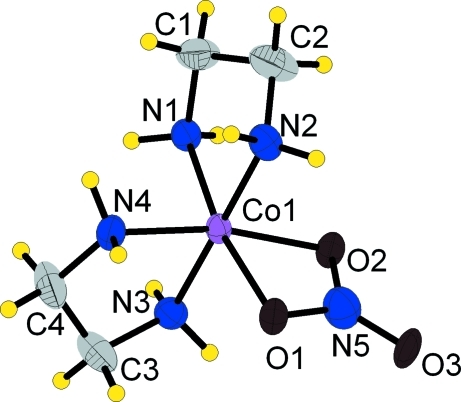
A view of (I). Displacement ellipsoids are drawn at the 50% probability level.

## supplementary crystallographic information

### Comment

Recently, a lot of diethylenediamine chelated Co(III) complexes which Cl^-^ or SO_4_^2-^ appeared as the second ligands have been reported (Niederhoffer *et al.*, 1986; Anderson *et al.*, 1977; Sharma *et al.*, 2006*a*,*b*,*c*). However, nitrite coordinated diethylenediamine chelated Co(III) complexes have not been reported. In this work, a new diethylenediamine chelated Co(III) complexes coordinated by a nitrite have been synthesized, and its structure is reported here.

The structure of the cation is given in Fig. 1. There are two crystallographically independent molecules in the asymmetric unit. The two molecules are almost identical. The cation consists of cobalt(III) coordinated by four nitrogen atoms from two ethylenediamine and two oxygen atoms from one nitrite. There is also a hydroxide and a nitrite appeared as the counter ions in the crystal. The Co—N and Co—O distances are normal (Sharma *et al.*, 2005, Bruggemann *et al.*, 1994).

In the crystal structure of title compound, there is strongly multipoint directional hydrogen bonds interactions among the [Co(en)_2_(NO_3_)] ^2+^ subunit, hydroxide and nitrite anions in the range of 0.278 (3) nm-0.3424 (12) nm. Thus, the complex cations and the hydroxide and nitrite anions are connected to result in a three-dimensional supramolecular framework through O–H···O and N–H···O hydrogen-bonding interactions.

### Experimental

The K_6_ H_2_[Nb_6_O_19_](H_2_O)_13_ oxidant (0.15 g, 0.11 mmol), Co(NO_3_)_2_ (0.15 g, 0.8 mmol) and 0.5 ml en in water (15 ml) was stirred for a hour at 80 °C. The resulting solution was filtered. Purple single crystals were obtained by slow evaporation of the filtrate at room temperature (yield 56% based on Co).

### Refinement

All H-atoms bound to carbon were refined using a riding model with d(C—H) = 0.93 Å, *U*_iso_ = 1.2*U*_eq_ (C). The imino H atoms were located in a difference Fourier map and refined isotropically with *U*_iso_(H) = 1.2 *U*_eq_(N). The hydroxy H atoms were also located in a difference Fourier map and refined isotropically with *U*_iso_(H) = 1.5 *U*_eq_(O).

### Crystal data

**Table:**

[Co(NO_3_)(C_2_H_8_N_2_)_2_](OH)(NO_3_)	*F*(000) = 1328
*M**_r_* = 320.17	*D*_x_ = 1.735 Mg m^−^^3^
Monoclinic, *P*2_1_/*c*	Mo *K*α radiation, λ = 0.71073 Å
Hall symbol: -P 2yn	Cell parameters from 4566 reflections
*a* = 9.5212 (13) Å	θ = 1.8–27.5°
*b* = 23.163 (3) Å	µ = 1.44 mm^−^^1^
*c* = 12.6473 (13) Å	*T* = 296 K
β = 118.491 (7)°	Block, purple
*V* = 2451.4 (5) Å^3^	0.38 × 0.34 × 0.28 mm
*Z* = 8	

### Data collection

**Table:**

Bruker APEX CCD area-detector diffractometer	4801 independent reflections
Radiation source: fine-focus sealed tube	3864 reflections with *I* > 2σ(*I*)
graphite	*R*_int_ = 0.080
ω scans	θ_max_ = 26.0°, θ_min_ = 2.0°
Absorption correction: multi-scan (*SADABS*; Sheldrick, 1996)	*h* = −5→11
*T*_min_ = 0.585, *T*_max_ = 0.660	*k* = −28→28
13327 measured reflections	*l* = −15→15

### Refinement

**Table:**

Refinement on *F*^2^	Primary atom site location: structure-invariant direct methods
Least-squares matrix: full	Secondary atom site location: difference Fourier map
*R*[*F*^2^ > 2σ(*F*^2^)] = 0.043	Hydrogen site location: inferred from neighbouring sites
*wR*(*F*^2^) = 0.116	H-atom parameters constrained
*S* = 1.00	*w* = 1/[σ^2^(*F*_o_^2^) + (0.0685*P*)^2^] where *P* = (*F*_o_^2^ + 2*F*_c_^2^)/3
4801 reflections	(Δ/σ)_max_ < 0.001
379 parameters	Δρ_max_ = 0.72 e Å^−^^3^
156 restraints	Δρ_min_ = −0.94 e Å^−^^3^

### Special details

**Table:**

Geometry. All e.s.d.'s (except the e.s.d. in the dihedral angle between two l.s. planes) are estimated using the full covariance matrix. The cell e.s.d.'s are taken into account individually in the estimation of e.s.d.'s in distances, angles and torsion angles; correlations between e.s.d.'s in cell parameters are only used when they are defined by crystal symmetry. An approximate (isotropic) treatment of cell e.s.d.'s is used for estimating e.s.d.'s involving l.s. planes.
Refinement. Refinement of *F*^2^ against ALL reflections. The weighted *R*-factor *wR* and goodness of fit *S* are based on *F*^2^, conventional *R*-factors *R* are based on *F*, with *F* set to zero for negative *F*^2^. The threshold expression of *F*^2^ > σ(*F*^2^) is used only for calculating *R*-factors(gt) *etc*. and is not relevant to the choice of reflections for refinement. *R*-factors based on *F*^2^ are statistically about twice as large as those based on *F*, and *R*- factors based on ALL data will be even larger.

### Fractional atomic coordinates and isotropic or equivalent isotropic displacement parameters (Å^2^)

**Table:**

	*x*	*y*	*z*	*U*_iso_*/*U*_eq_	Occ. (<1)
Co1	0.63400 (4)	0.342196 (16)	0.24054 (3)	0.02089 (13)	
Co2	0.63022 (5)	0.091878 (15)	0.34954 (3)	0.02108 (13)	
O1	0.6097 (3)	0.26004 (9)	0.23269 (19)	0.0282 (5)	
O2	0.6617 (3)	0.31005 (8)	0.11093 (19)	0.0285 (5)	
O3	0.6478 (3)	0.21277 (9)	0.0920 (2)	0.0383 (6)	
O4	0.7145 (2)	0.05508 (8)	0.50374 (18)	0.0247 (4)	
O5	0.5824 (2)	0.01077 (8)	0.33416 (18)	0.0259 (5)	
O6	0.6690 (3)	−0.04038 (9)	0.5060 (2)	0.0349 (5)	
O7	0.3076 (11)	0.2225 (4)	0.3737 (11)	0.078 (3)	0.65
O8	0.083 (2)	0.2623 (16)	0.2871 (12)	0.089 (9)	0.30
O9	0.207 (3)	0.2545 (12)	0.4756 (10)	0.080 (7)	0.50
O10	0.7596 (13)	0.5293 (3)	0.6156 (7)	0.068 (2)	0.80
O11	0.6248 (9)	0.4956 (4)	0.4388 (4)	0.0504 (18)	0.75
O12	0.737 (2)	0.4389 (3)	0.5867 (12)	0.057 (3)	0.70
O7A	0.265 (2)	0.2069 (7)	0.3600 (18)	0.087 (7)	0.35
O8A	0.0835 (10)	0.2683 (6)	0.2866 (7)	0.063 (3)	0.70
O9A	0.184 (2)	0.2457 (11)	0.4693 (9)	0.061 (3)	0.50
O10A	0.795 (5)	0.5268 (11)	0.616 (2)	0.078 (10)	0.20
O11A	0.674 (3)	0.4977 (14)	0.4408 (10)	0.065 (7)	0.25
O12A	0.768 (6)	0.4390 (7)	0.582 (3)	0.064 (10)	0.30
N1	0.6761 (3)	0.42325 (10)	0.2250 (2)	0.0261 (5)	
H1A	0.6611	0.4301	0.1502	0.031*	
H1B	0.6077	0.4456	0.2371	0.031*	
N2	0.8638 (3)	0.33570 (11)	0.3456 (2)	0.0311 (6)	
H2A	0.8852	0.3339	0.4229	0.037*	
H2B	0.9008	0.3033	0.3281	0.037*	
N3	0.4046 (3)	0.35017 (11)	0.1353 (2)	0.0292 (6)	
H3A	0.3823	0.3855	0.1017	0.035*	
H3B	0.3706	0.3237	0.0762	0.035*	
N4	0.5871 (3)	0.35552 (11)	0.3722 (2)	0.0292 (6)	
H4A	0.6075	0.3232	0.4168	0.035*	
H4B	0.6502	0.3839	0.4199	0.035*	
N5	0.6397 (4)	0.25792 (13)	0.1409 (3)	0.0435 (7)	
N6	0.8303 (3)	0.07897 (11)	0.3447 (2)	0.0293 (6)	
H5A	0.8087	0.0661	0.2714	0.035*	
H5B	0.8884	0.0519	0.3991	0.035*	
N7	0.7121 (3)	0.16859 (10)	0.4071 (2)	0.0289 (6)	
H6A	0.6870	0.1793	0.4644	0.035*	
H6B	0.6684	0.1941	0.3463	0.035*	
N8	0.4281 (3)	0.10719 (11)	0.3472 (2)	0.0308 (6)	
H7A	0.4303	0.1423	0.3783	0.037*	
H7B	0.4108	0.0807	0.3918	0.037*	
N9	0.5185 (3)	0.11287 (11)	0.1795 (2)	0.0302 (6)	
H8A	0.5063	0.0814	0.1343	0.036*	
H8B	0.5769	0.1388	0.1641	0.036*	
N10	0.6554 (3)	0.00506 (12)	0.4507 (3)	0.0394 (7)	
N11	0.1917 (4)	0.24459 (12)	0.3766 (3)	0.0401 (7)	
N12	0.7160 (3)	0.48825 (12)	0.5470 (3)	0.0370 (7)	
C1	0.8423 (4)	0.43773 (14)	0.3142 (4)	0.0423 (9)	
H1C	0.8793	0.4707	0.2871	0.051*	
H1D	0.8484	0.4472	0.3910	0.051*	
C2	0.9419 (4)	0.38679 (16)	0.3266 (4)	0.0518 (11)	
H2C	1.0478	0.3918	0.3945	0.062*	
H2D	0.9524	0.3819	0.2546	0.062*	
C3	0.3235 (5)	0.34185 (18)	0.2100 (4)	0.0482 (10)	
H3C	0.2159	0.3573	0.1686	0.058*	
H3D	0.3170	0.3010	0.2243	0.058*	
C4	0.4164 (5)	0.37191 (19)	0.3238 (4)	0.0502 (10)	
H4C	0.3792	0.3612	0.3803	0.060*	
H4D	0.4041	0.4133	0.3113	0.060*	
C5	0.8883 (4)	0.16771 (14)	0.4572 (3)	0.0376 (8)	
H5C	0.9385	0.1499	0.5362	0.045*	
H5D	0.9290	0.2067	0.4646	0.045*	
C6	0.9235 (4)	0.13326 (15)	0.3715 (3)	0.0400 (8)	
H6C	0.8928	0.1549	0.2981	0.048*	
H6D	1.0368	0.1248	0.4078	0.048*	
C7	0.2979 (4)	0.10441 (16)	0.2198 (3)	0.0405 (8)	
H7C	0.2010	0.1221	0.2118	0.049*	
H7D	0.2751	0.0647	0.1927	0.049*	
C8	0.3603 (4)	0.13742 (16)	0.1477 (3)	0.0409 (9)	
H8C	0.2885	0.1331	0.0622	0.049*	
H8D	0.3697	0.1782	0.1676	0.049*	
O14	−0.0621 (4)	0.18855 (15)	0.0907 (3)	0.0656 (9)	
H14	0.0071	0.2147	0.1342	0.079*	
O13	0.9458 (4)	0.54280 (13)	0.8748 (3)	0.0680 (9)	
H13	0.9031	0.5301	0.7909	0.082*	

### Atomic displacement parameters (Å^2^)

**Table:**

	*U*^11^	*U*^22^	*U*^33^	*U*^12^	*U*^13^	*U*^23^
Co1	0.0252 (2)	0.0169 (2)	0.0252 (2)	0.00281 (15)	0.01582 (18)	0.00258 (15)
Co2	0.0280 (2)	0.0156 (2)	0.0247 (2)	−0.00050 (15)	0.01659 (18)	0.00059 (15)
O1	0.0430 (13)	0.0187 (10)	0.0342 (12)	0.0029 (9)	0.0277 (11)	0.0043 (8)
O2	0.0451 (13)	0.0188 (10)	0.0326 (12)	0.0001 (9)	0.0274 (10)	0.0018 (9)
O3	0.0585 (16)	0.0190 (11)	0.0524 (15)	0.0003 (10)	0.0387 (13)	−0.0096 (10)
O4	0.0348 (12)	0.0168 (10)	0.0254 (10)	−0.0026 (8)	0.0166 (9)	−0.0009 (8)
O5	0.0329 (11)	0.0188 (10)	0.0247 (11)	−0.0028 (8)	0.0125 (9)	−0.0012 (8)
O6	0.0497 (14)	0.0169 (10)	0.0436 (14)	−0.0006 (9)	0.0267 (12)	0.0101 (9)
O7	0.060 (4)	0.051 (5)	0.133 (6)	0.026 (4)	0.054 (4)	0.009 (4)
O8	0.077 (14)	0.085 (16)	0.055 (9)	0.031 (11)	−0.009 (10)	−0.023 (10)
O9	0.110 (13)	0.074 (10)	0.046 (5)	−0.012 (8)	0.029 (6)	−0.019 (5)
O10	0.059 (5)	0.063 (3)	0.079 (4)	−0.009 (3)	0.030 (3)	−0.038 (3)
O11	0.059 (4)	0.055 (3)	0.036 (2)	0.010 (3)	0.022 (2)	0.010 (2)
O12	0.086 (6)	0.041 (3)	0.045 (4)	−0.004 (3)	0.033 (4)	0.009 (3)
O7A	0.086 (12)	0.039 (8)	0.145 (13)	0.027 (8)	0.061 (10)	−0.010 (8)
O8A	0.060 (5)	0.074 (6)	0.061 (5)	0.034 (4)	0.033 (4)	0.024 (5)
O9A	0.065 (6)	0.063 (8)	0.059 (6)	−0.011 (6)	0.033 (5)	0.009 (6)
O10A	0.050 (17)	0.061 (12)	0.116 (16)	−0.045 (13)	0.034 (13)	−0.030 (13)
O11A	0.095 (17)	0.076 (11)	0.064 (8)	0.025 (11)	0.071 (9)	0.035 (7)
O12A	0.11 (2)	0.044 (8)	0.045 (10)	0.027 (10)	0.038 (11)	0.009 (7)
N1	0.0287 (13)	0.0211 (12)	0.0323 (14)	0.0023 (10)	0.0176 (11)	0.0031 (11)
N2	0.0299 (14)	0.0304 (14)	0.0348 (15)	0.0058 (11)	0.0168 (12)	0.0069 (11)
N3	0.0283 (14)	0.0258 (14)	0.0339 (15)	0.0003 (10)	0.0152 (12)	0.0012 (11)
N4	0.0378 (15)	0.0238 (13)	0.0329 (14)	0.0016 (11)	0.0225 (12)	−0.0004 (11)
N5	0.0473 (19)	0.0405 (17)	0.0500 (19)	0.0032 (14)	0.0293 (15)	0.0046 (14)
N6	0.0341 (15)	0.0282 (13)	0.0320 (14)	−0.0008 (11)	0.0209 (12)	−0.0016 (11)
N7	0.0461 (16)	0.0170 (12)	0.0296 (14)	0.0001 (11)	0.0229 (13)	0.0023 (10)
N8	0.0387 (15)	0.0260 (13)	0.0386 (15)	0.0060 (11)	0.0273 (13)	0.0061 (11)
N9	0.0389 (16)	0.0279 (14)	0.0262 (14)	−0.0041 (11)	0.0175 (12)	0.0014 (11)
N10	0.0416 (17)	0.0329 (16)	0.0528 (19)	−0.0020 (12)	0.0298 (15)	−0.0047 (13)
N11	0.0412 (17)	0.0253 (15)	0.0535 (19)	0.0038 (13)	0.0224 (15)	0.0017 (14)
N12	0.0438 (17)	0.0335 (16)	0.0456 (17)	−0.0023 (13)	0.0310 (15)	−0.0034 (13)
C1	0.0336 (19)	0.0314 (18)	0.056 (2)	−0.0060 (14)	0.0162 (17)	−0.0015 (16)
C2	0.0319 (19)	0.044 (2)	0.075 (3)	−0.0017 (16)	0.0223 (19)	0.011 (2)
C3	0.0321 (19)	0.066 (3)	0.054 (2)	−0.0009 (17)	0.0268 (18)	0.0038 (19)
C4	0.044 (2)	0.065 (3)	0.056 (2)	0.0075 (19)	0.036 (2)	−0.002 (2)
C5	0.042 (2)	0.0274 (17)	0.042 (2)	−0.0103 (14)	0.0186 (16)	−0.0045 (15)
C6	0.0352 (19)	0.0390 (19)	0.052 (2)	−0.0078 (15)	0.0256 (17)	0.0018 (17)
C7	0.0306 (18)	0.0392 (19)	0.051 (2)	0.0031 (15)	0.0192 (16)	0.0080 (17)
C8	0.038 (2)	0.040 (2)	0.038 (2)	0.0047 (15)	0.0129 (16)	0.0072 (16)
O14	0.0562 (19)	0.094 (2)	0.0485 (17)	−0.0051 (16)	0.0264 (15)	−0.0153 (16)
O13	0.062 (2)	0.066 (2)	0.072 (2)	−0.0089 (15)	0.0289 (17)	−0.0057 (16)

### Geometric parameters (Å, °)

**Table:**

Co1—O1	1.914 (2)	N3—H3A	0.8998
Co1—O2	1.930 (2)	N3—H3B	0.8999
Co1—N4	1.944 (3)	N4—C4	1.487 (4)
Co1—N1	1.949 (2)	N4—H4A	0.9000
Co1—N2	1.950 (3)	N4—H4B	0.9001
Co1—N3	1.950 (3)	N6—C6	1.482 (4)
Co1—N5	2.338 (3)	N6—H5A	0.9000
Co2—O4	1.919 (2)	N6—H5B	0.9001
Co2—O5	1.921 (2)	N7—C5	1.484 (4)
Co2—N7	1.937 (2)	N7—H6A	0.9002
Co2—N8	1.943 (3)	N7—H6B	0.9004
Co2—N9	1.952 (3)	N8—C7	1.494 (4)
Co2—N6	1.958 (3)	N8—H7A	0.9000
Co2—N10	2.334 (3)	N8—H7B	0.9000
O1—N5	1.322 (4)	N9—C8	1.475 (4)
O2—N5	1.312 (3)	N9—H8A	0.8999
O3—N5	1.236 (4)	N9—H8B	0.9001
O4—N10	1.323 (3)	C1—C2	1.474 (5)
O5—N10	1.302 (3)	C1—H1C	0.9700
O6—N10	1.236 (4)	C1—H1D	0.9700
O7—N11	1.233 (6)	C2—H2C	0.9700
O8—N11	1.183 (8)	C2—H2D	0.9700
O9—N11	1.212 (8)	C3—C4	1.458 (6)
O10—N12	1.219 (5)	C3—H3C	0.9700
O11—N12	1.233 (5)	C3—H3D	0.9700
O12—N12	1.227 (6)	C4—H4C	0.9700
O7A—N11	1.199 (8)	C4—H4D	0.9700
O8A—N11	1.241 (5)	C5—C6	1.507 (5)
O9A—N11	1.210 (7)	C5—H5C	0.9700
O10A—N12	1.225 (9)	C5—H5D	0.9700
O11A—N12	1.226 (9)	C6—H6C	0.9700
O12A—N12	1.236 (10)	C6—H6D	0.9700
N1—C1	1.479 (4)	C7—C8	1.512 (5)
N1—H1A	0.9000	C7—H7C	0.9700
N1—H1B	0.9000	C7—H7D	0.9700
N2—C2	1.476 (4)	C8—H8C	0.9700
N2—H2A	0.8999	C8—H8D	0.9700
N2—H2B	0.8996	O14—H14	0.8708
N3—C3	1.489 (5)	O13—H13	0.9823
			
O1—Co1—O2	68.55 (8)	Co2—N8—H7B	110.0
O1—Co1—N4	97.52 (10)	H7A—N8—H7B	108.3
O2—Co1—N4	165.82 (10)	C8—N9—Co2	110.2 (2)
O1—Co1—N1	167.98 (10)	C8—N9—H8A	109.5
O2—Co1—N1	99.67 (10)	Co2—N9—H8A	109.6
N4—Co1—N1	94.35 (11)	C8—N9—H8B	109.8
O1—Co1—N2	91.56 (10)	Co2—N9—H8B	109.6
O2—Co1—N2	89.15 (11)	H8A—N9—H8B	108.1
N4—Co1—N2	94.04 (12)	O6—N10—O5	125.6 (3)
N1—Co1—N2	85.71 (10)	O6—N10—O4	123.7 (3)
O1—Co1—N3	89.45 (10)	O5—N10—O4	110.7 (3)
O2—Co1—N3	91.12 (11)	O6—N10—Co2	178.9 (2)
N4—Co1—N3	85.94 (11)	O5—N10—Co2	55.40 (14)
N1—Co1—N3	93.28 (10)	O4—N10—Co2	55.30 (14)
N2—Co1—N3	178.98 (11)	O8—N11—O7A	113.6 (18)
O1—Co1—N5	34.43 (10)	O8—N11—O9A	119.3 (15)
O2—Co1—N5	34.14 (9)	O7A—N11—O9A	119.8 (9)
N4—Co1—N5	131.93 (11)	O8—N11—O9	122.8 (9)
N1—Co1—N5	133.72 (11)	O7A—N11—O9	121.8 (17)
N2—Co1—N5	89.75 (11)	O9A—N11—O9	13 (3)
N3—Co1—N5	91.02 (11)	O8—N11—O7	120.0 (9)
O4—Co2—O5	68.44 (8)	O7A—N11—O7	24.0 (14)
O4—Co2—N7	97.30 (10)	O9A—N11—O7	120.6 (12)
O5—Co2—N7	165.56 (10)	O9—N11—O7	116.3 (8)
O4—Co2—N8	91.59 (10)	O8—N11—O8A	6(3)
O5—Co2—N8	89.52 (10)	O7A—N11—O8A	117.4 (9)
N7—Co2—N8	93.24 (11)	O9A—N11—O8A	118.1 (7)
O4—Co2—N9	167.03 (10)	O9—N11—O8A	120.1 (12)
O5—Co2—N9	98.85 (10)	O7—N11—O8A	121.3 (8)
N7—Co2—N9	95.50 (11)	O10—N12—O10A	16 (3)
N8—Co2—N9	85.56 (11)	O10—N12—O11A	117.6 (18)
O4—Co2—N6	91.29 (10)	O10A—N12—O11A	115.0 (12)
O5—Co2—N6	92.00 (10)	O10—N12—O12	120.2 (6)
N7—Co2—N6	85.91 (11)	O10A—N12—O12	118 (2)
N8—Co2—N6	177.08 (11)	O11A—N12—O12	121 (2)
N9—Co2—N6	91.74 (11)	O10—N12—O11	120.2 (5)
O4—Co2—N10	34.52 (9)	O10A—N12—O11	124.0 (19)
O5—Co2—N10	33.92 (9)	O11A—N12—O11	21.7 (13)
N7—Co2—N10	131.79 (11)	O12—N12—O11	118.3 (6)
N8—Co2—N10	90.43 (10)	O10—N12—O12A	121.6 (19)
N9—Co2—N10	132.70 (11)	O10A—N12—O12A	114.5 (12)
N6—Co2—N10	92.22 (11)	O11A—N12—O12A	114.9 (12)
N5—O1—Co1	90.63 (17)	O12—N12—O12A	15 (3)
N5—O2—Co1	90.25 (18)	O11—N12—O12A	118.1 (19)
N10—O4—Co2	90.18 (17)	C2—C1—N1	107.4 (3)
N10—O5—Co2	90.68 (17)	C2—C1—H1C	110.2
C1—N1—Co1	109.76 (19)	N1—C1—H1C	110.2
C1—N1—H1A	109.7	C2—C1—H1D	110.2
Co1—N1—H1A	109.7	N1—C1—H1D	110.2
C1—N1—H1B	109.7	H1C—C1—H1D	108.5
Co1—N1—H1B	109.7	C1—C2—N2	108.1 (3)
H1A—N1—H1B	108.2	C1—C2—H2C	110.1
C2—N2—Co1	108.4 (2)	N2—C2—H2C	110.1
C2—N2—H2A	110.1	C1—C2—H2D	110.1
Co1—N2—H2A	110.0	N2—C2—H2D	110.1
C2—N2—H2B	110.1	H2C—C2—H2D	108.4
Co1—N2—H2B	109.9	C4—C3—N3	108.1 (3)
H2A—N2—H2B	108.4	C4—C3—H3C	110.1
C3—N3—Co1	107.8 (2)	N3—C3—H3C	110.1
C3—N3—H3A	109.9	C4—C3—H3D	110.1
Co1—N3—H3A	110.1	N3—C3—H3D	110.1
C3—N3—H3B	110.4	H3C—C3—H3D	108.4
Co1—N3—H3B	110.1	C3—C4—N4	108.3 (3)
H3A—N3—H3B	108.4	C3—C4—H4C	110.0
C4—N4—Co1	109.9 (2)	N4—C4—H4C	110.0
C4—N4—H4A	109.7	C3—C4—H4D	110.0
Co1—N4—H4A	109.6	N4—C4—H4D	110.0
C4—N4—H4B	109.8	H4C—C4—H4D	108.4
Co1—N4—H4B	109.7	N7—C5—C6	107.1 (3)
H4A—N4—H4B	108.2	N7—C5—H5C	110.3
O3—N5—O2	125.2 (3)	C6—C5—H5C	110.3
O3—N5—O1	124.2 (3)	N7—C5—H5D	110.3
O2—N5—O1	110.5 (3)	C6—C5—H5D	110.3
O3—N5—Co1	177.6 (3)	H5C—C5—H5D	108.5
O2—N5—Co1	55.62 (15)	N6—C6—C5	107.4 (3)
O1—N5—Co1	54.94 (15)	N6—C6—H6C	110.2
C6—N6—Co2	110.4 (2)	C5—C6—H6C	110.2
C6—N6—H5A	109.6	N6—C6—H6D	110.2
Co2—N6—H5A	109.6	C5—C6—H6D	110.2
C6—N6—H5B	109.5	H6C—C6—H6D	108.5
Co2—N6—H5B	109.6	N8—C7—C8	105.4 (3)
H5A—N6—H5B	108.1	N8—C7—H7C	110.7
C5—N7—Co2	108.56 (18)	C8—C7—H7C	110.7
C5—N7—H6A	109.9	N8—C7—H7D	110.7
Co2—N7—H6A	110.0	C8—C7—H7D	110.7
C5—N7—H6B	109.9	H7C—C7—H7D	108.8
Co2—N7—H6B	110.0	N9—C8—C7	106.5 (3)
H6A—N7—H6B	108.3	N9—C8—H8C	110.4
C7—N8—Co2	108.4 (2)	C7—C8—H8C	110.4
C7—N8—H7A	110.2	N9—C8—H8D	110.4
Co2—N8—H7A	110.0	C7—C8—H8D	110.4
C7—N8—H7B	109.9	H8C—C8—H8D	108.6

### Hydrogen-bond geometry (Å, °)

**Table:**

*D*—H···*A*	*D*—H	H···*A*	*D*···*A*	*D*—H···*A*
O13—H13···O10	0.98	1.97	2.906 (8)	158
O14—H14···O8	0.87	2.04	2.78 (3)	143
N1—H1A···O4^i^	0.90	2.17	3.032 (3)	160
N1—H1B···O5^ii^	0.90	2.19	2.995 (3)	148
N2—H2A···O14^iii^	0.90	2.00	2.888 (4)	168
N2—H2B···O8^iv^	0.90	2.25	3.045 (19)	148
N3—H3A···O6^ii^	0.90	2.10	2.986 (3)	169
N3—H3B···O9^i^	0.90	2.33	3.14 (2)	150
N3—H3B···O7^i^	0.90	2.57	3.424 (12)	159
N4—H4A···O3^v^	0.90	2.22	3.004 (3)	145
N4—H4B···O11	0.90	2.62	3.328 (10)	136
N4—H4B···O12	0.90	2.26	3.071 (14)	150
N6—H5A···O12^i^	0.90	2.10	2.973 (14)	163
N6—H5B···O13^i^	0.90	2.32	2.985 (4)	131
N6—H5B···O13^vi^	0.90	2.53	3.258 (4)	138
N7—H6A···O2^v^	0.90	1.99	2.881 (3)	168
N7—H6B···O1	0.90	1.98	2.873 (3)	169
N8—H7A···O7	0.90	2.18	2.988 (7)	149
N8—H7B···O6^vii^	0.90	2.01	2.887 (3)	163
N9—H8A···O11^viii^	0.90	2.29	3.088 (9)	147
N9—H8A···O12^i^	0.90	2.58	3.08 (2)	115
N9—H8B···O3	0.90	2.19	3.065 (3)	163

Symmetry codes: (i) *x*, −*y*+1/2, *z*−1/2; (ii) −*x*+1, *y*+1/2, −*z*+1/2; (iii) *x*+1, −*y*+1/2, *z*+1/2; (iv) *x*+1, *y*, *z*; (v) *x*, −*y*+1/2, *z*+1/2; (vi) −*x*+2, *y*−1/2, −*z*+3/2; (vii) −*x*+1, −*y*, −*z*+1; (viii) −*x*+1, *y*−1/2, −*z*+1/2.
